# Integrated care: a comprehensive bibliometric analysis and literature review

**Published:** 2014-06-12

**Authors:** Xiaowei Sun, Wenxi Tang, Ting Ye, Yan Zhang, Bo Wen, Liang Zhang

**Affiliations:** Research Centre of Rural Healthcare Services, School of Medicine and Health Management, Tongji Medical College, Huazhong University of Science & Technology, Wuhan, China; Research Centre of Rural Healthcare Services, School of Medicine and Health Management, Tongji Medical College, Huazhong University of Science & Technology, Wuhan, China; Research Centre of Rural Healthcare Services, Tongji Medical College, Huazhong University of Science & Technology, Wuhan, China; Research Centre of Rural Healthcare Services, School of Medicine and Health Management, Tongji Medical College, Huazhong University of Science & Technology, Wuhan, China; Tongji Hospital, Tongji Medical College, Huazhong University of Science & Technology, Wuhan, China; Research Centre of Rural Healthcare Services, School of Medicine and Health Management, Tongji Medical College, Huazhong University of Science & Technology, Wuhan, China

**Keywords:** integrated care, bibliometric analysis, delivery of health care, integrated, literature review

## Abstract

**Introduction:**

Integrated care could not only fix up fragmented health care but also improve the continuity of care and the quality of life. Despite the volume and variety of publications, little is known about how ‘integrated care’ has developed. There is a need for a systematic bibliometric analysis on studying the important features of the integrated care literature.

**Aim:**

To investigate the growth pattern, core journals and jurisdictions and identify the key research domains of integrated care.

**Methods:**

We searched Medline/PubMed using the search strategy ‘(delivery of health care, integrated [MeSH Terms]) OR integrated care [Title/Abstract]’ without time and language limits. Second, we extracted the publishing year, journals, jurisdictions and keywords of the retrieved articles. Finally, descriptive statistical analysis by the Bibliographic Item Co-occurrence Matrix Builder and hierarchical clustering by SPSS were used.

**Results:**

As many as 9090 articles were retrieved. Results included: (1) the cumulative numbers of the publications on integrated care rose perpendicularly after 1993; (2) all documents were recorded by 1646 kinds of journals. There were 28 core journals; (3) the USA is the predominant publishing country; and (4) there are six key domains including: the definition/models of integrated care, interdisciplinary patient care team, disease management for chronically ill patients, types of health care organizations and policy, information system integration and legislation/jurisprudence.

**Discussion and conclusion:**

Integrated care literature has been most evident in developed countries. *International Journal of Integrated Care* is highly recommended in this research area. The bibliometric analysis and identification of publication hotspots provides researchers and practitioners with core target journals, as well as an overview of the field for further research in integrated care.

## Introduction

Because of the ageing of the population, improvement of people's health consciousness and fast-growing medical scientific knowledge [1], the fragmentation of health care services is increasingly serious. As a result, neither the country nor person can afford the seriously growing disease burden. In addition, the health delivery system is one of the typical cases of complex adaptive systems [2]. What physicians and patients face is not only a medical problem but also a social problem with the development of bio-psycho-social medical models [3].Thus the health delivery system should deliver comprehensive and continuous care (integrated care as we discuss in this paper) combined with health risk factors prevention, diagnosis, treatment and rehabilitation.

Integrated care has been widely discussed all over the world. Even though health care seems too complex for a one-size-fits-all integrated care delivery system [4], integrated care seems to have significantly positive effects on the delivery and utilization of health care [5–7]. Evidence shows that integrated care not only can fix up fragmented health care but also effectively reduce hospitalization, emergency room, average length of stay and health expenditure [8, 9], while improving the quality of life [10]. Some systematic reviews on integrated care were reported [6, 11–14] which discussed integrated care in the aspect of health economics, implementation and patients with chronic conditions, but there was no article analysis by bibliometrics. Studying the important features of the integrated care literature, especially the growth pattern, journal productivity, jurisdictions and key research fields, is significant.

Bibliometrics is the quantitative study of literature as reflected in bibliographies, which provides insight into the growth of literature and how research findings are disseminated to readers of journals in a specified field of academic research. This is becoming increasingly important in the evidence-based practice movement [15]. This method provides evolutionary models of science, technology and scholarship. The most prominent model for distribution of bibliographic items is Bradford's law and Zipf's law. Bradford proposed the concepts of core and scatter. Core refers to the small number of journals that publish the most papers in a field; scatter refers to the spread of literature over many publications. Zipf's law refers to the distribution of keywords that a word frequency is inversely proportional to its ranking in the frequency table. Beyond this, the bibliometric method can identify topical key research domains by hierarchical clustering related topics.

The main purpose of this study intends to accomplish the following objectives:

1. investigate the growth pattern of the integrated care literature;
2. find the jurisdiction distribution of the integrated care literature;
3. identify the core journals that contains a substantial proportion of the total integrated care literature and investigate the features of these nucleus journals; and
4. identify the key research domains of integrated care and describe the key elements of each domain.

## Materials and methods

### Literature search

Electronic database: Medline/PubMed.

Research strategy: (delivery of health care, integrated [MeSH Terms]) OR integrated care [Title/Abstract], update to October 16, 2013, no language limit.

### Analysis methods

The papers retrieved were analyzed by the Bibliographic Item Co-occurrence Matrix Builder [16]. This software, which was adopted as a way to manage a large number of data to analysis the trends, identify the core journals and calculate the keywords co-occurrence matrix, has been used in several studies including cancer nursing [17] and chronic disease self-management [18]. After we searched the literature, we extracted the publishing time, journals, jurisdictions and keywords of the literature by Bibliographic Item Co-occurrence Matrix Builder. Then the analysis was separated into two steps: (1) a descriptive statistics analysis on the growth pattern, language, core journals and jurisdictions of the publications; and (2) a hierarchical clustering analysis on the keywords by using the software SPSS after the keywords co-occurrence matrix analysis by Bibliographic Item Co-occurrence Matrix Builder.

## Results

### The descriptive statistics analysis

#### Literature growth

**Figure 1.**
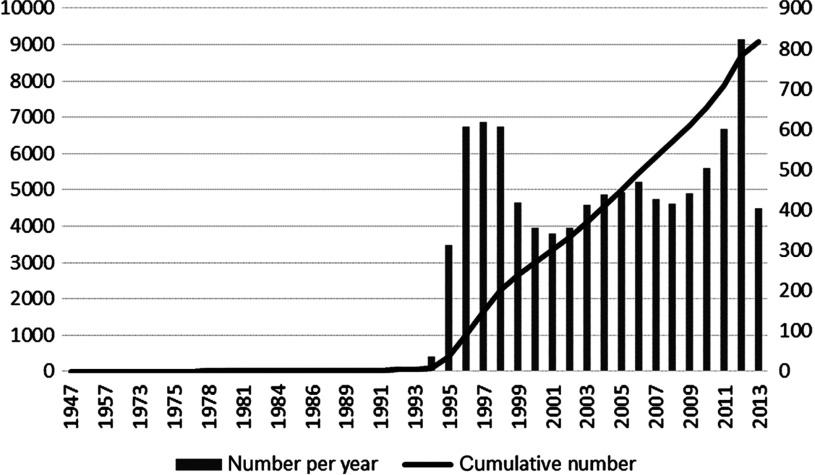
Numbers and cumulative numbers of articles on integrated care in PubMed, 1947–2013

As many as 9090 articles were retrieved for the entire study period. The line chart shows the cumulative numbers of the relative articles, while the bar chart shows the numbers of relative articles per year (Figure 1). The first article about integrated care ‘Architectural plan for integrated care’ was written by Sarvis [19] in 1947, recorded by the journal Hospitals, which was not available on the internet. As it shows, there was a long period of time with publishing less than 10 articles per year till 1992, and then the cumulative numbers of the articles rose perpendicularly. During the years 1995 to 1998, there was a perpendicular increase in publications on integrated care from 16 publications per year to 617 publications per year. Afterwards over 300 articles on integrated care were reported every year. During 1998 to 2009, the number of publications was fluctuating up and down at the level of 400 publications per year.

#### Language of publications

The primary language was English (92.90%), followed by German (3.49%), French (0.89%), Spanish (0.57%), Portuguese (0.51%) and others (1.64%).

#### Core journals of publications

All of those articles were recorded by 1646 kinds of journals. Of these, 696 journals have published only one paper on integrated care. Based on the Bradford hypothesis, we divided all the journals into four zones by quartile of the cumulative number. The nucleus of journals (zone 1) consists of 28 journals, followed by 121 titles (zone 2), 379 titles (zone 3) and 1267 titles (zone 4). The ratio of journal number among these four zones is 28:93:286:1267 = 1:3.32:10.21:45.25, which is quite close to 1:3.3:3.3^2^(10.89):3.3^3^(35.937). Table 1 shows the 28 core journals in descending order, numbers of articles and cumulative percentage, impact factor, publication frequency and country, and subject field. *Modern Healthcare* (*Mod Healthc*), *Healthcare Financial Management: Journal of Healthcare Financial Management Association* (*Healthc Financ Manage*) and *International Journal of Integrated Care* (*Int J Integr Care*, IF = 1.299) are the first three journals.

Two of the 28 core journals have changed name. Medical Network Strategy Report changed its name to Physician Performance and Payment Report, while Health System Leader changed its name to Executive Solutions for Health care Management. Twenty-two of 28 journals are published in the USA, followed by England (4), Canada (1) and Netherlands (1). Only 5 of 28 journals are recorded by Science Citation Index and only 1 journal recorded by Social Science Citation Index, which was the *International Journal of Integrated Care*. The highest IF in 2012 was the journal *Health Affairs* (Project Hope; 4.641), followed by *American Journal of Public Health* (3.93). Although the core integrated care literature is concentrated into a small number of journals, the journals are quite diversified in their subject coverage. The publications of the journals are weekly (3), monthly (11), bimonthly (4), quarterly (4) and others (6).

#### Jurisdictions of the retrieved documents

**Figure 2.**
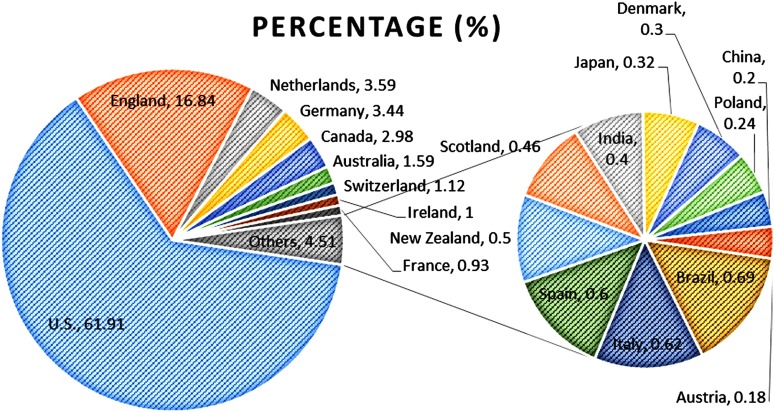
Pie graph of the top 20 countries ranked by the percentage of publications

Figure 2 shows the pie graph of the top 20 countries ranked by the percentage of publications. The USA is the country with the largest results output, with a total of 5598, accounting for 61.91% of the total literature. While England had reached a total of 1523, with a percentage of 16.84 compared with the USA. The cumulative percentage of 20 countries reached up to 97.9%. The 80% concentration ratio (countries covering 80% of the documents) is 2, while 90% of it is 6. North America (USA and Canada) was by far the most productive area in the field of integrated care, responsible for 66.27% of all articles, followed by 29.95% from Europe (UK, Netherlands, Germany, Switzerland, Ireland, France, Italy, Spain, Scotland, Denmark, Poland and Austria). There are rare articles written by researchers from developing countries (India and China, 0.60%).

#### Core keywords of publications

Retrieved articles contained a sum of 5875 keywords, while 2961 keywords only appeared once. We also divided the keywords into four zones to identify the core keyword so as to analysis by hierarchical cluster. The nucleus of keywords (zone 1) consists of 41 keywords (Table 2), followed by 177 keywords (zone 2), 804 keywords (zone 3) and 4870 keywords (zone 4). The ratio of journal number among these four zones is 41:177:804:4870 = 1:4.3:19.6:118.8, which is quite close to 1:4.3:4.3^2^(18.49):4.3^3^(79.51), except for zone 4, which is even larger than the prediction.

#### Hierarchical cluster analysis results

**Figure 3.**
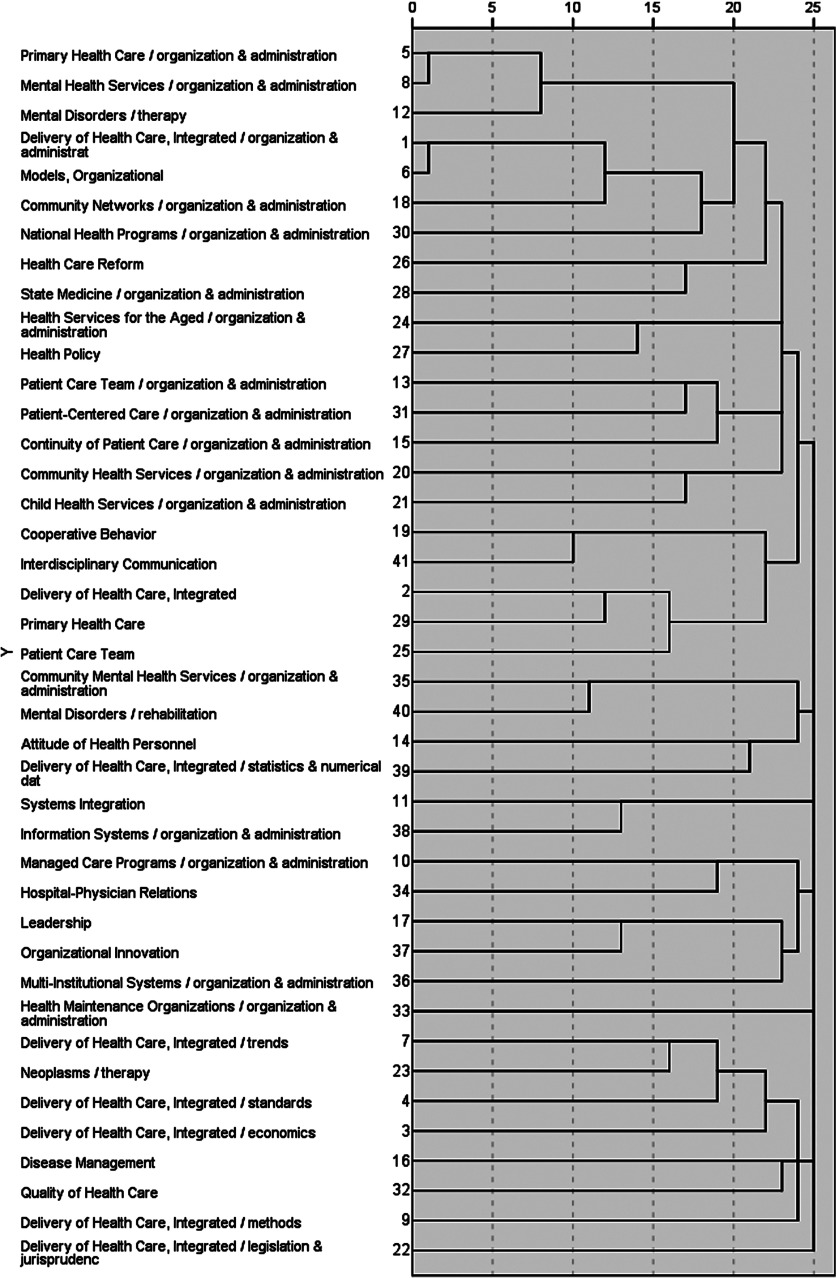
The tree diagram of the hierarchical cluster analysis on the 41 keywords

The core keywords co-occurrence matrices were analyzed by SPSS. Figure 3 shows the tree diagram result of hierarchical cluster analysis on 41 core keywords. After our research team discussed, as the tree diagram will not show an optimal cluster result automatically, the six key domains of integrated care (Table 3) were as follows:

Definitions and conceptual models of integrated care: the concept and characteristics of integrated care had been widely discussed and variously used.

Interdisciplinary patient care team: the variety of reasons for the need of interdisciplinary team work, fundamental concepts and features associated with team work and the characteristics underpinning effective interdisciplinary team work.

Disease management for chronically ill patients: the derivation, development and the feature of disease management, the two typical cases of disease management (i.e. Kaiser Permanente [20] and Chronic Care Model [21]).

Types of integrated health care delivery organizations and policy: new concepts of integrated care organizations and policy developed to improving care coordination and outcomes, reducing fragmental care and costs.

Information system integration: a design of an information system with the functions to share and provide the comprehensive picture of the safety and quality of health care, and improve the outcomes.

Legislation/jurisprudence on health care: the need for the health care act to improve quality, patient safety and cost-effectiveness.

## Discussion

The curve of the publications on integrated care seems to be fluctuating up and down more than that observed in other bibliometric analyses, such as Randomized Controlled Trial [22], primary care [23], especially there was a sudden jump from 1995. To investigate whether there were any artificial reasons, we analyzed the including time by PubMed of journals which published papers in 1995. The 109 kinds of journals issued the 313 articles, while 17 of those were newly included in PubMed and published 80 articles in 1995, only 25.56% of the total. More importantly, the sudden interest of the academic research on integrated care seemed to be the reason. In the 1990s, the integrated delivery systems were set up to focus on better care co-ordination as a means of improving quality and reducing cost, even though most of these systems failed to deliver savings^24^. Then an integrated (or organized) delivery system, the first notion similar to integrated care, was described in 1994 by Schortell et al. [25]. Furthermore, an extensive discussion about the different definition, concepts of integrated delivery between the USA and the other countries, mostly in the Europe, was developed [26–29]. Afterwards, the MeSH term ‘Delivery of health system, integrated’, which was the first and the only standard MeSH term on integrated care, was introduced in 1996 based on the definition by Coddington et al. [30].

There is no doubt that English is the main language of integrated care research output, followed by German and French, because MEDLINE is a US-based database and English is the official language. Nucleus journals usually contain articles with the highest impact in the area and, thus, subscriptions to such journals in indexing and abstracting services would be justified scientifically [31]. The top journal was *International Journal of Integrated Care*, established in 2000 and cited by Social Science Citation Index, with the mission of promoting integrated care as a scientific discipline. Moreover, *International Journal of Integrated Care* is the only journal whose primary purpose is to examine critically the policy and practice of integrated care and whether and how this has impacted on quality-of-care, user experiences and cost-effectiveness. Unsurprisingly, the USA is the predominant publishing country in the integrated care (approximately 78.57% of the journals and 61.91% of the articles). Clearly, publications in integrated care research are related to the country's degree of development.

The result of the cluster analysis of frequently used by keywords extracted from publications directly describes the research status and challenge of the research on integrated care. First, even though there is no universally accepted definition of integrated and no one-size-fits-all model or process for successful integration, researchers can understand the concepts and features of integrated care differently, including: the perspective of definition [29, 32–34], the framework of integrated care [35–37], the different classification for integrated care (types, breadth, degree and process [38]) and the difference within the terms related to integrated care [39]. Esther Suter et al. [4] identified 10 key principles for successful health system integration and defined key areas for restructuring and allowing organizational flexibility and adaptation to local context, but there is not a firm empirical foundation for specific integration strategies and processes that can be replicated locally [40].

Second, the interdisciplinary patient care team was the integrated care provider in various types of health care organizations related to integrated care. The increasing need for integration was caused by (1) an ageing population with frail older people and larger numbers of patients with more complex needs associated with chronic diseases; (2) the increasing complexity of skills and knowledge required to provide comprehensive care to patients; and (3) increasing specialization within health professions and a corresponding fragmentation of disciplinary knowledge resulting in no-one health care professional being able to meet all the complex needs of their patients [41]. In order to overcome those difficulties, the features and characteristics underpinning effective interdisciplinary team work [42–44], with the typical disease management model and various types of health care organizations and policy, had been issued. Besides, an integrated framework for the management of safety, quality and risk is needed, with an information and incident management system based on a universal patient safety classification [45].

The Kaiser Permanente and Chronic Care Model seemed to be the best choice of the physicians to best manage the patients with complex chronic conditions. One of those key features taken by Kaiser Permanente is the application of a population management (or care) model that divides the insured population of patients with chronic conditions into three distinct groups based on their degree of need [46], which had become known as the Kaiser Permanente ‘triangle’ or ‘pyramid of care’. While the Chronic Care Model [47, 48] was characterized by productive interactions between the four key parts: practice team and patients, involving assessment, self-management support, optimization of therapy and follow-up.

More and more integrated approaches to care delivery are required to improve the quality, patient experience and reduce the health cost. Those reforms have encompassed and often combined a range of organizational (health maintenance organizations, accountable care organizations), financial (bundling, health-saving accounts s, pay for performance, accountable care organizations) and informational (health information technology, comparative effectiveness research) approaches. Accountable care organizations were propelled forward by the 2010 Patient Protection and Affordable Care Act as the solution while integrated delivery system and health maintenance organizations have morphed into accountable care organizations [49, 50]. However, the accountable care organizations have also faced a number of challenges and should not be seen as a ‘magic bullet’ [51]. In order to control costs effectively researchers and policy-makers should pay more attention to what other countries do to slow down health care spending. Global budgets, fee schedules, system wide payment rules and concentrated purchasing power may not be modern, exciting or transformational, but have the advantage of working.

Information system integration was the foundation of the integrated care system. The integrated framework should include the conventional medical record and ancillary information about patients, investigations and procedures, a system for logging, managing and monitoring progress when things go wrong, a data repository for collating information from all available sources, and a risk management framework underpinning both proactive and reactive responses [45].

Last, a specific health care act was the best choice to accomplish several goals at once for integration. However, there are four key challenges that currently prevent the best joined-up care, including predominance of small group practices, dominant fee-for-service reimbursement methods, weaknesses of the traditional hospital medical staff structure and a need to partner with commercial insurance companies [52]. In any case, an act or plan on integrated care as a component of care in legislation should include at least two objectives: (1) to improve quality, patient safety and cost-effectiveness; and (2) detailing how the programmes can work synergistically, and how best practices in finance and payment, in the organization and delivery of care and in prevention, can be expanded nationally [53].

## Conclusion

Integrated care has increasingly attracted public concern from the year 1994, especially in developed countries. *International Journal of Integrated Care* is highly recommended in this research area among the 28 core journals. The research area concerns several disciplines and the research domain mainly focuses on better understanding the features and characteristics of integrated care and several strategies to effectively apply integrated care and replicate locally. However, the mechanism within the several strategies requires more discussion.

Although the bibliometric method has some limitations, the results nevertheless demonstrate that it is a useful tool for identifying the preponderance of research in one area. It can guide the researchers and health care policy makers to be more focused in planning and organizing research in the field of integrated care.

## Figures and Tables

**Table 1.**
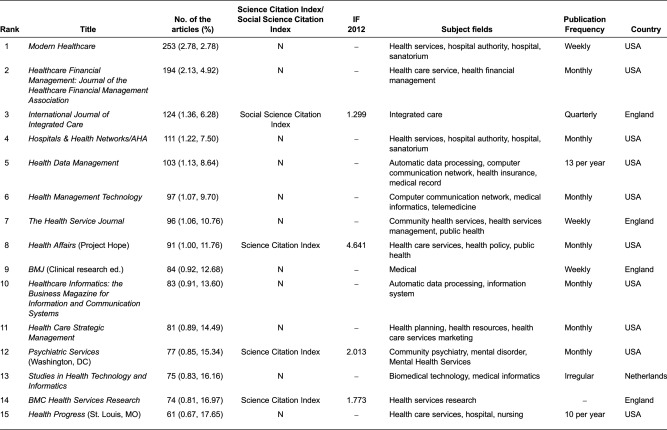

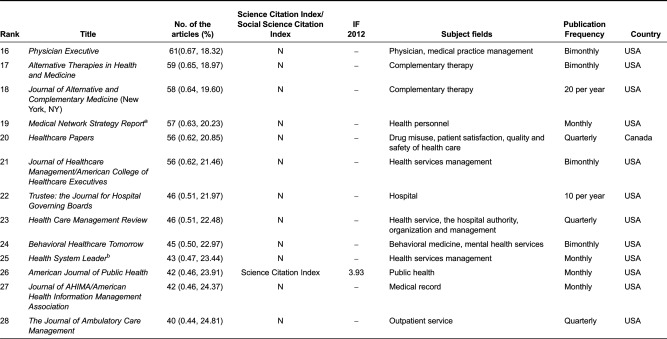
Numbers of journal articles, impact factors, subject fields and publication frequency and country of 28 core journals

^a^Title changed to Physician performance & payment report.

^b^Title changed to Executive solutions for health care management.

**Table 2.**
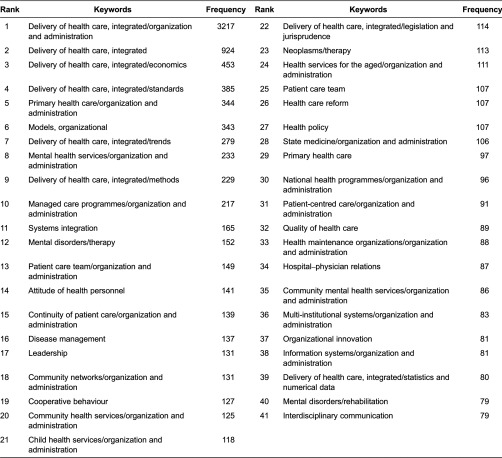
The 41 keywords of the publications on integrated care

**Table 3.**
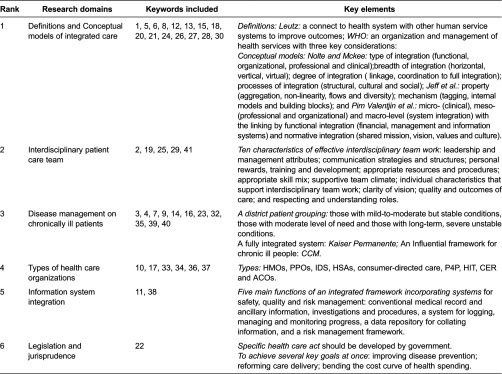
Key elements of the six research domains of integrated care

Abbreviations: HMOs, health maintenance organizations; PPOs, preferred provider organizations; IDS, integrated delivery system; HSAs, health-saving accounts; P4P, pay for performance; HIT, health information technology; CER, comparative effectiveness research; ACOs, accountable care organizations; CCM, Chronic Care Model.
